# Acute Severity Versus Long-term Morbidity: Uncoupling the Roles of RSV and HRV in Childhood Respiratory Disease

**DOI:** 10.1093/ofid/ofag314

**Published:** 2026-05-21

**Authors:** Lu Qin, Na Ling, Xin-Qin Feng, Wei Li, Xiang-Zhi Wang, Lan-Fang Tang

**Affiliations:** Department of Pulmonology, Children's Hospital, Zhejiang University School of Medicine, National Clinical Research Center for Children and Adolescents’ Health and Diseases, Hangzhou, China; Department of Pulmonology, Children's Hospital, Zhejiang University School of Medicine, National Clinical Research Center for Children and Adolescents’ Health and Diseases, Hangzhou, China; Department of Pulmonology, Children's Hospital, Zhejiang University School of Medicine, National Clinical Research Center for Children and Adolescents’ Health and Diseases, Hangzhou, China; Department of Clinical Laboratory, The Children's Hospital of Zhejiang University School of Medicine, Hangzhou, Zhejiang Province, China; Department of Pulmonology, Children's Hospital, Zhejiang University School of Medicine, National Clinical Research Center for Children and Adolescents’ Health and Diseases, Hangzhou, China; Department of Pulmonology, Children's Hospital, Zhejiang University School of Medicine, National Clinical Research Center for Children and Adolescents’ Health and Diseases, Hangzhou, China

**Keywords:** asthma, eosinophil, human rhinovirus, immunity, respiratory syncytial virus

## Abstract

**Background:**

The distinct roles of respiratory syncytial virus (RSV) and human rhinovirus (HRV) in acute illness and subsequent asthma remain debated. China's strict nonpharmaceutical interventions (NPIs) in 2022 provided a unique epidemiological setting to compare their clinical/inflammatory profiles and associations with long-term asthma risk.

**Methods:**

This observational retrospective cohort study included children hospitalized with RSV- or HRV-induced acute lower respiratory tract infections. We collected clinical data, subtyped RSV samples and conducted long-term follow-up to assess physician-diagnosed asthma using Kaplan–Meier and multivariable logistic regression.

**Results:**

Among 722 children (425 RSV, 275 HRV, and 22 coinfection), RSV predominated in infants with a sharp winter peak (RSV-A dominant, 96.8%), while HRV circulated year-round across a broader age range. RSV infection was associated with greater acute severity, including higher rates of pneumonia and longer hospitalization (*P* < .05). In contrast, HRV triggered a distinct inflammatory response with significant leukocytosis and an eosinophil count over 4-fold greater than the RSV group (*P* < .001). Follow-up revealed HRV infection was independently associated with increased risk of subsequent asthma (log-rank *P* < .001). Multivariable regression identified prior wheezing (aOR 2.82, 95% CI: 1.76–4.53), elevated eosinophils (aOR 2.43, 95% CI: 1.07–5.49), and HRV infection (aOR 1.85, 95% CI: 1.15–2.99) as independent predictors for asthma.

**Conclusions:**

During China's late NPI phase, HRV infection showed a stronger independent association with childhood asthma than RSV. This association, together with an eosinophil-predominant inflammatory profile, remained significant after adjustment for prior wheezing history. These findings should be interpreted as associations rather than evidence of causality.

Acute lower respiratory tract infections (ALRTIs) are a leading cause of pediatric hospitalization worldwide [1], with respiratory syncytial virus (RSV) and human rhinovirus (HRV) being the key pathogens precipitating first-time wheezing episodes [2, 3]. RSV is a member of the *Pneumovirus* genus in the *Paramyxoviridae* family and was historically recognized as the primary cause of severe bronchiolitis in infants, leading to a substantial disease burden globally [4, 5]. HRV belongs to the Enterovirus genus in the *Picornaviridae* family and is traditionally associated with the common cold; it is increasingly acknowledged as a significant contributor to wheezing illnesses and asthma exacerbations in children of all ages [6, 7]. Despite their high prevalence, the distinct roles of RSV and HRV in shaping both acute clinical presentation and long-term respiratory morbidity remain a subject of ongoing investigation.

During the COVID-19 pandemic, the incidence of these respiratory viral infections markedly decreased because of widespread nonpharmaceutical interventions (NPIs) [8]. However, following the relaxation of these measures, the resurgence of respiratory viruses has been observed worldwide. This “immunity debt” phenomenon has led to potentially increased disease severity due to a lack of immune stimulation in the pediatric population [9]. In China, strict public health measures have been maintained longer than in many other regions, creating a unique epidemiological landscape. The year 2022 represented a critical transition period characterized by fluctuating control measures and eventual relaxation. Although the increasing trend of respiratory infections was interrupted during the peak of the COVID-19 restrictions, a marked rebound in ALRTIs was noticed in clinical practice during the second half of 2022 [10]. Unlike the predictable seasonal patterns of the prepandemic era, this resurgence presented unique challenges to the healthcare system [11]. Understanding the specific contributions of RSV and HRV during this unique period is therefore crucial.

Although both viruses cause similar clinical syndromes, RSV and HRV are thought to trigger distinct host immune responses with disparate long-term consequences [12]. RSV infection is characterized by airway epithelial necrosis and mixed inflammatory cell infiltration, including neutrophils and lymphocytes, leading to small airway obstruction [13]. HRV infection, by contrast, has been associated with a distinct inflammatory profile involving eosinophilic activation and type 2 cytokine responses in susceptible hosts, although the precise mechanisms remain under investigation [14]. A particular concern is the link between early life infection and the development of asthma. While RSV is a known risk factor, increasing evidence suggests that HRV, especially in children with atopic predisposition or eosinophilia, might be a stronger predictor of future asthma [15]. In addition, viral genetic diversity contributes to disease complexity. For RSV, the circulation of specific subtypes may influence clinical severity, yet data regarding the dominant strains during 2022 in China remain limited.

Understanding the reasons behind the differential clinical profiles and long-term risks of respiratory viral resurgence in 2022 will help inform national prevention and control strategies. Thus, this retrospective cohort study of children hospitalized with ALRTIs in 2022 was designed to characterize the viral distribution and predominant RSV subtypes, compare the clinical and inflammatory profiles of RSV and HRV infections, and ultimately determine the independent association of each virus with the long-term risk of asthma and recurrent wheezing.

## METHODS

### Study Design and Data Source

This retrospective study was performed at the Children's Hospital of Zhejiang University School of Medicine. We reviewed the laboratory records of all patients diagnosed with ALRTIs and hospitalized children aged <17 years who tested positive for RSV or HRV between January 1, 2022, and December 31, 2022. ALRTIs were defined as the presence of signs and symptoms of respiratory tract infection (ie, fever, coughing, rhinorrhea, oropharyngeal hyperemia, swelling of tonsils) and lower respiratory signs (tachypnea, dyspnea, retractions, or wheezing/rales upon auscultation) [16]. Nasopharyngeal swab samples were collected within 24 hours of admission and preserved in viral transport medium (KaiBiLi, Hangzhou, China). Nucleic acids were extracted using an automated system (Health, Ningbo, China). Respiratory pathogen detection was performed using a multiplex fluorescence PCR kit coupled with capillary electrophoresis (Health, Ningbo, China). The multiplex respiratory pathogen panel included RSV, HRV, influenza A and B viruses, human parainfluenza virus, adenovirus, human metapneumovirus, human bocavirus, coronavirus, Mycoplasma pneumoniae, and Chlamydophila pneumoniae. Amplified products were separated using a CE2400 capillary electrophoresis instrument (Juno Genomics, Hangzhou, China). Pathogen positivity was determined by comparing the specific loci peak heights with standard controls. Because the present study was specifically designed to evaluate the clinical characteristics and follow-up outcomes of children with RSV and/or HRV infection, only records of patients with RSV and/or HRV positivity were extracted into the analytic data-set. Although routine clinical testing covered a broader respiratory pathogen panel, the present study was restricted to children with RSV and/or HRV positivity.

### Study Population and Grouping

The source population consisted of 2393 unique hospitalized children who tested positive for RSV, HRV, or both viruses during 2022. From this source population, a cohort was selected for detailed clinical analysis and long-term follow-up based on the following inclusion criteria: (1) had no history of physician-diagnosed asthma prior to the current episode of ALRTI; (2) had laboratory-confirmed infection with a single RSV, single HRV, or coinfection with both viruses; and (3) had complete clinical data and follow-up records sufficient to determine the development of asthma or recurrent wheezing. Patients presenting with any of the following conditions were excluded: (1) primary or secondary immunodeficiency; (2) congenital heart disease; (3) congenital airway malformations or pulmonary dysplasia; (4) airway foreign body aspiration; (5) severe systemic comorbidities, including sepsis, solid tumors, or leukemia; or (6) a history of pulmonary trauma. The final study cohort was divided into 3 groups according to viral etiology: the RSV group, the HRV group, and the coinfection group. A comparison of available baseline characteristics between the source population and the final analytic cohort is presented in Supplementary Table 1.

### Clinical Data and Follow-up

Baseline demographics, clinical symptoms, radiological findings, and laboratory parameters (blood counts and biochemistry) were extracted from electronic medical records. Telephone follow-up was conducted to assess the subsequent development of wheezing episodes and asthma in childhood in November 2025. Personal atopic history was defined as the presence of at least one of the following conditions documented in the medical record or reported by the caregiver: allergic rhinitis (AR), eczema, atopic dermatitis (AD), allergic conjunctivitis, urticaria, drug or food allergy, or positive results for specific allergens (including house dust mites, mold, animal dander, and pollen) on previous allergen testing. Family atopic history was defined as the presence of at least one of the following conditions in a first-degree relative: AR, eczema, AD, allergic conjunctivitis, allergic asthma, urticaria, or drug or food allergy. The primary outcome was the development of physician-diagnosed asthma during the follow-up period, defined as “ever asthma” (ie, a new asthma diagnosis made at any point during the follow-up by a pediatrician). Asthma was diagnosed according to the criteria of the Chinese Pediatric Society guidelines for childhood bronchial asthma (2016 revision) [17]. Secondary outcomes included the occurrence of recurrent wheezing, defined as 3 or more parent-reported wheezing episodes within any 12-month period during follow-up [18]. The complete follow-up questionnaire used at each time point is provided in Supplementary 2.

### Viral RNA Extraction and RSV Typing

A subset of 250 nasopharyngeal swab samples, confirmed as RSV-positive by initial screening, was randomly selected for subtyping. Total viral nucleic acids were extracted via an automated nucleic acid extraction system (EX3600, Shanghai ZJ Bio-Tech Co., Ltd., Shanghai, China) according to the manufacturer's instructions. Reverse transcription (RT) was performed to synthesize cDNA via a standard cDNA synthesis kit (HiScript III All-in-one RT SuperMix for qPCR; Vazyme Biotech Co., Ltd., Nanjing, China). The resulting cDNA served as the template for subsequent PCR amplification.

Genotyping was carried out via specific primers targeting the N protein regions of RSV-A and RSV-B (RSVA-NF 5'-CTCAATTTCCTCACTTCTC' and RSVA-NR 5'-CTTGATTCCTCGGTGTACCTGTGT-3' and RSVB-NF 5'-TTCCTAACTTCTCAAGTGTGGTCCTA-3' and RSVB-GR 5'-CTOGGTTTCTTGGCGTACCTCTATAC-3'). The PCR mixture contained 5 μL of master mix, 0.5 μL of each primer, and 1 µL of cDNA template. The cycling conditions were as follows: initial denaturation at 94°C for 5 minutes, followed by 28 cycles of denaturation at 94°C for 30 seconds, annealing at 60°C for 30 seconds, and extension at 72°C for 30 seconds, with a final extension at 72°C for 2 minutes.

The PCR products were separated via 2% agarose gel electrophoresis and visualized under ultraviolet light. RSV-A and RSV-B were distinguished on the basis of the presence of specific amplification bands. Samples with low viral loads that failed to amplify during subtyping were recorded as untypeable.

### Statistical Analysis

Continuous variables are presented as medians (interquartile ranges, IQRs) and were compared using the Mann–Whitney *U* test or the Kruskal–Wallis test. Categorical variables were expressed as frequencies and percentages and were analyzed by the χ^2^ test. Epidemiological trends were visualized by plotting case numbers against age and calendar month. Kaplan–Meier curves were used to estimate asthma-free survival. Multivariable logistic regression models were used to identify factors independently associated with asthma, adjusting for potential confounders such as age, atopy, and eosinophil counts. All analyses were performed via SPSS 26.0 and GraphPad Prism 9.0, with a two-sided *P* value < .05 indicating statistical significance.

## RESULTS

### Epidemiological Features of the Overall Surveillance Population

**Figure 1.**
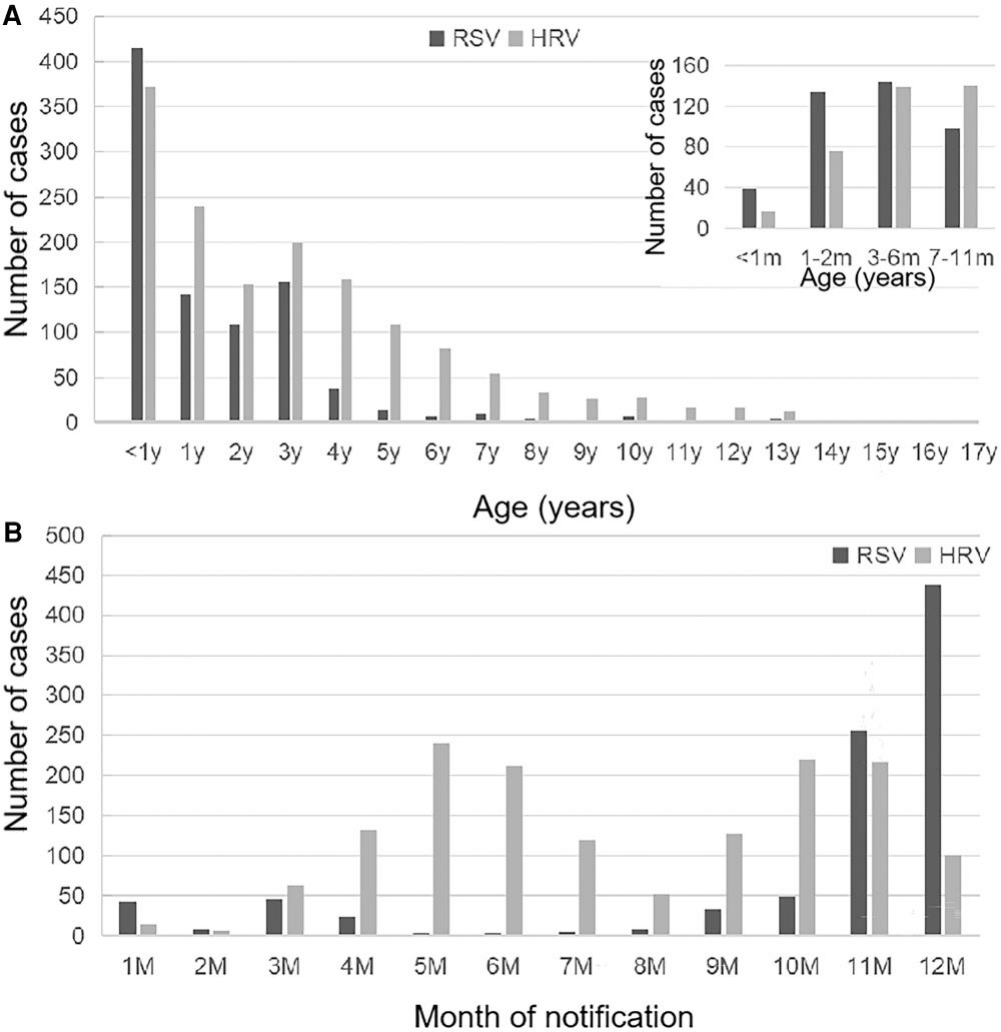
Epidemiological characteristics of hospitalized children with RSV and HRV infections in 2022. *A*, Age distribution of RSV- and HRV-positive patients. The majority of RSV cases occurred in infants under 1 y of age, whereas HRV cases exhibited a broader age distribution. The inset chart details the age distribution for infants under 12 m. *B*, Seasonal distribution of confirmed cases by calendar month. RSV infection showed a dominant peak in winter (November and December), whereas HRV infection presented year-round activity, with peaks in spring (May) and autumn (October).

In 2022, a total of 2393 hospitalized children tested positive for either RSV or HRV. Specifically, RSV was detected in 914 patients, accounting for 12.9% of all virus-positive hospitalizations (914/7110), whereas HRV was identified in 1507 patients, accounting for 21.2% of the total (1507/7110). The overall ratio of male to female patients was 1.39:1 for RSV (532 vs 382) and 1.60:1 for HRV (928 vs 579). Significant differences were observed in the age distributions of the 2 viruses (Figure 1*A*).

RSV infection predominantly affected infants, with 45.4% (415/914) of all cases occurring in children under 1 year of age. Following infancy, the number of RSV cases declined sharply. Conversely, HRV infection demonstrated a broader age distribution. While also common in infants (24.7%, 372/1507), HRV was the leading cause of infection among toddlers and preschool children aged 1–5 years, accounting for 57.1% (860/1507) of the total cases. This age disparity was also reflected in our final study cohort, in which the median age of the HRV group was significantly greater than that of the RSV group (*P* < .001), as shown in Table 1.

**Table 1.** Baseline Demographic and Clinical Characteristics of the Study Population

	RSV (425)	HRV (275)	RSV + HRV (22)	*P* Value
Age (yr)	0.77 (2.71)	1.67 (2.76)	0.37 (0.83)	<.001
Gender, *n* (%) male/female				.092
Male/female	248 (58.4%)	180 (65.5%)	16 (72.7%)	
Female	177 (41.6%)	95 (34.5%)	6 (27.3%)	
Weight (kg)	9.50 (7.35)	11.50 (6.50)	8.15 (4.03)	<.001
Birth history, *n* (%)				.993
Term	365 (85.9%)	237 (86.2%)	19 (86.4%)	
Preterm	60 (14.1%)	38 (13.8%)	3 (13.6%)	
Feeding, *n* (%)				.222
Breast milk	256 (60.2%)	152 (55.3%)	10 (45.5%)	
Artificial milk	51 (12.0%)	40 (14.5%)	6 (27.3%)	
Mixed	118 (27.8%)	83 (30.2%)	6 (27.3%)	
Atopic history, *n* (%)	78 (18.4%)	73 (26.5%)	4 (18.2%)	.035
Family atopic history, *n* (%)	39 (9.2%)	49 (17.8%)	4 (18.2%)	.003
History of wheezing, *n* (%)	78 (18.4%)	102 (37.1%)	7 (31.8%)	<.001

Abbreviations: IQR, interquartile range; kg, kilogram.

The occurrence of RSV and HRV infections also displayed distinct seasonal patterns (Figure 1*B*). RSV activity was minimal during the first half of the year but increased dramatically from November to December, coinciding with the winter season. In contrast, HRV circulated year-round and exhibited a bimodal distribution, with a primary peak in late spring (May) and a secondary peak in autumn (October).

Routine clinical testing in our center covered a broader respiratory pathogen panel; however, the present analysis focused exclusively on children with RSV and/or HRV positivity.

### Baseline Characteristics of the Study Cohort

After applying the inclusion and exclusion criteria, 722 patients were included in the final analysis (RSV group: *n* = 425; HRV group: *n* = 275; coinfection group: *n* = 22). A comparison between the source population and the analytic cohort showed that while the sex distribution remained highly consistent, the final cohort had a lower median age (1.08 vs 2.08 years) and a higher proportion of RSV infections (Supplementary Table 1). This expected demographic shift reflects our strict exclusion criteria, particularly the exclusion of children with prior physician-diagnosed asthma, which predominantly filtered out older children with HRV infections. Consistent with the overall epidemiological data, the median age of the HRV group was significantly greater than that of the RSV group (1.67 vs 0.77 years, *P* < .001) (Table 1). Furthermore, children with HRV had a significantly greater prevalence of personal atopic history (26.5% vs 18.4%, *P* = .035), family history of atopy (17.8% vs 9.2%, *P* = .003), and history of wheezing prior to admission (37.1% vs 18.4%, *P* < .001).

### Clinical Phenotypes and Virological Features

Important clinical features are shown in Table 2. The length of hospitalization was significantly different between the groups (*P* = .009), with the RSV group having a slightly longer median stay (5.0 days) than the HRV group did (4.0 days). Fever was a more common feature in the RSV group (61.9%) than in the HRV group (47.6%), and the duration of fever was also longer in RSV patients (*P* < .001). Despite similar rates of wheezing on physical examination (63.3% vs 62.2%, *P* = .612), the duration of cough was significantly longer in the RSV group (median 10.0 days vs 8.0 days, *P* < .001). Chest X-ray findings also differed, with pneumonia being the most common finding in RSV patients (39.1%), whereas bronchitis was more common in the HRV group (38.9%) (*P* = .002).

**Table 2.** Clinical Severity and Laboratory Findings Among the 3 Groups

	RSV (425)	HRV (275)	RSV + HRV (22)	*P* Value
Clinical Severity and Treatment				
Fever, *n* (%)	263 (61.9%)	131 (47.6%)	10 (45.5%)	<.001
Duration of fever (d) Median (IQR)	2.00 (5.0)	0.00 (3.0)	0.00 (2.0)	<.001
Duration of coughing (d) Median (IQR)	10.00 (4.0)	8.00 (5.0)	10.00 (4.0)	<.001
Wheezing *n* (%)	269 (63.3%)	171 (62.2%)	16 (72.7%)	.612
Oxygen support, *n* (%)	100 (23.5%)	82 (29.8%)	3 (13.6%)	.075
Systemic steroid use, *n* (%)	181 (42.6%)	131 (47.6%)	7 (31.8%)	.209
Duration of systemic steroid use (d) Median (IQR)	0.00 (3.0)	0.00 (3.0)	0.00 (3.0)	.473
Length of hospitalization (d) Median (IQR)	5.00 (2.0)	4.00 (3.0)	5.00 (3.0)	.009
Chest X-ray, *n* (%)				.002
Bronchitis	121 (28.5%)	107 (38.9%)	6 (27.3%)	
Bronchopneumonia	138 (32.5%)	98 (35.6%)	6 (27.3%)	
Pneumonia	166 (39.1%)	70 (25.5%)	10 (45.5%)	
Laboratory ﬁndings				
WBC (×10^9^/L) median (IQR)	7.31 (3.7)	12.93 (6.11)	9.83 (12.2)	<.001
LYC (×10^9^/L) median (IQR)	2.70 (2.2)	3.44 (2.9)	4.22 (4.7)	.048
Eos (×10^9^/L) median (IQR)	0.04 (0.1)	0.18 (0.3)	0.09 (0.1)	<.001
Hb (g/L) Median (IQR)	121.00 (12.0)	125.50 (13.5)	126.00 (14.0)	.007
PLT (×10^9^/L) Median (IQR)	272.00 (120.0)	346.50 (134.0)	404.00 (299.0)	<.001
CRP (mg/dL) Median (IQR)	4.53 (11.5)	7.82 (12.8)	0.70 (8.3)	.04
IgE (IU/mL) Median (IQR)	85.35 (169.2)	109.50(226.2)	91.80 (176.7)	.361
IgG (g/L) Median (IQR)	7.74 (3.3)	7.45 (2.9)	7.35 (2.2)	.337
IgA (g/L) Median (IQR)	0.56 (0.75)	0.49 (0.6)	0.57 (0.3)	.487
IgM (g/L) Median (IQR)	1.10 (0.6)	1.02 (0.5)	1.49 (1.0)	.188
ALT (U/L) Median (IQR)	17.00 (8.0)	14.50 (8.0)	24.00 (11.0)	<.001
AST (U/L) Median (IQR)	45.50 (24.8)	34.00 (13.0)	37.00 (36.0)	<.001
LDH (U/L) Median (IQR)	360.00 (174.0)	301.50 (101.8)	348.00 (126.0)	<.001
CKMB (U/L) Median (IQR)	26.50 (23.5)	24.00 (16.5)	32.00 (22.0)	.003

Abbreviations: IQR, interquartile range; WBC, white blood cell; LYC, lymphocyte count; Eos, eosinophil count; Hb, hemoglobin; PLT, platelet; CRP, C-reactive protein; Ig, immunoglobulin; ALT, glutamic-pyruvic transaminase; AST, glutamic oxalacetic transaminase; LDH, lactate dehydrogenase; CKMB, creatine kinase.

In addition, laboratory findings revealed distinct inflammatory profiles. Leukocytosis was more pronounced in the HRV group, which had a significantly greater median WBC count than the RSV group did (12.93 vs 7.31 × 10^9^/L, *P* < .001). Most notably, the HRV group exhibited significantly higher eosinophil counts than the RSV group, with a median value more than 4-fold greater (0.18 vs 0.04 × 10^9^/L, *P* < .001). Platelet counts were also significantly elevated in the HRV group (*P* < .001).

To explore potential virological drivers for these clinical observations, we performed subtyping on 250 randomly selected RSV-positive samples. Among the 155 successfully genotyped cases, RSV-A was the overwhelmingly predominant strain, accounting for 96.8% (150/155) of the infections. In contrast, RSV-B was identified in only a small fraction of the patients (3.2%, 5/155). The remaining 95 samples were untypeable, which was attributed to low viral loads in these retrospective specimens.

### Long-term Respiratory Outcomes and Risk Factor Analysis

**Figure 2.**
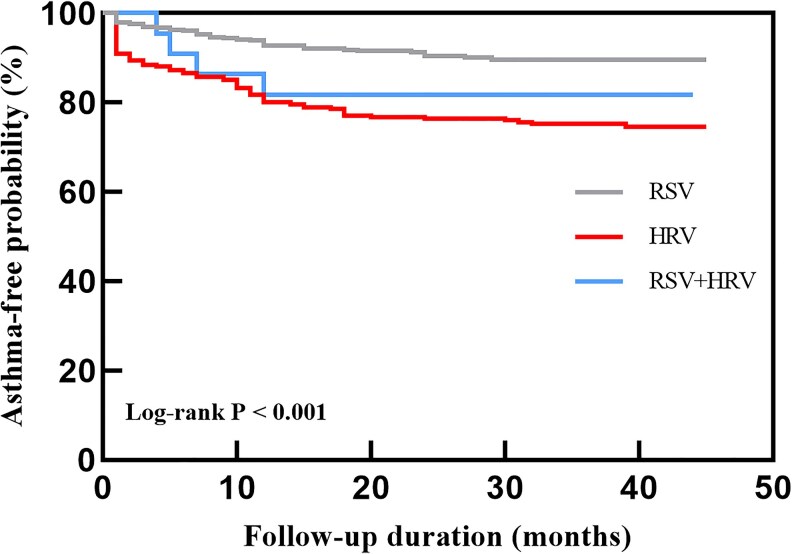
K–M survival curves for asthma diagnosis in different virus groups. The probability of asthma-free survival was monitored over the follow-up period. Compared with the RSV group (gray line) and the coinfection group (blue line), the HRV group (red line) presented a significantly greater cumulative incidence of asthma (log-rank *P* < .001).

All 722 patients were successfully followed up for a median duration of 36 months. During this period, 117 (16.20%) children were diagnosed with asthma at a median age of 2.84 years. Among these, 82 (70.1%) were diagnosed before 4 years of age, 27 (23.1%) between 4 and 5 years, and 8 (6.8%) at 6 years or older. Additionally, 148 (20.50%) children experienced recurrent wheezing. Kaplan–Meier survival analysis was used to assess the probability of asthma-free survival over time (Figure 2). The cumulative incidence of asthma was significantly different among the 3 groups (log-rank *P* < .001). Compared with the RSV and coinfection groups, the HRV group presented the lowest asthma-free survival rate, indicating a higher cumulative incidence of subsequent asthma.

To identify factors independently associated with these long-term outcomes while adjusting for baseline characteristics, we performed multivariable logistic regression analyses (Table 3). HRV infection was independently associated with both asthma diagnosis (aOR = 1.85, 95% CI: 1.15–2.99, *P* = .012) and recurrent wheezing (aOR = 1.85, 95% CI: 1.23–2.79, *P* = .003).

**Table 3.** Multivariate Logistic Regression Analysis of Factors Associated With Asthma Diagnosis and Recurrent Wheezing

	Asthma Diagnosis		Recurrent Wheezing	
Variables	aOR (95% CI)	*P* Value	aOR (95% CI)	*P* Value
HRV	1.85 (1.15–2.99)	.012	1.85 (1.23–2.79)	.003
RSV + HRV	1.24 (.33–4.69)	.751	1.43(.50–4.11)	.503
Age (years)	1.11 (.95–1.30)	.183	0.86 (.75–1.00)	.045
Gander (male)	1.30 (.81–2.10)	.281	1.26 (.84–1.91)	.268
Atopic	1.54 (.92–2.57)	.100	1.38(.85–2.24)	.200
Family history	1.32 (.73–2.40)	.359	1.20 (.69–2.08)	.526
History of wheezing	2.82 (1.76–4.53)	<.001	1.41 (.90–2.23)	.136
Eos count	2.43 (1.07–5.49)	.033	1.43 (.83–2.37)	.202

A *P* value of <.05 was considered statistically significant.

Abbreviations: aOR, adjusted odds ratio; CI, confidence interval.

Crucially, the regression models highlighted distinct risk profiles for asthma versus recurrent wheezing. A higher eosinophil count at the time of initial infection was independently associated with a subsequent asthma diagnosis (aOR = 2.43, 95% CI: 1.07–5.49, *P* = .033) but not with recurrent wheezing. Similarly, a prior history of wheezing was independently associated with asthma diagnosis (aOR = 2.82, 95% CI: 1.76–4.53, *P* < .001), underscoring the cumulative risk in susceptible individuals. In contrast, younger age emerged as a significant risk factor specifically for recurrent wheezing (aOR = 0.86, 95% CI: .75–1.00, *P* = .045), likely reflecting the anatomical vulnerability of the airways in younger infants, whereas personal atopic history was not statistically significant in the final adjusted models.

## DISCUSSION

In 2022, China maintained strict NPIs against COVID-19, creating a unique epidemiological environment with reduced exposure to common respiratory pathogens. Within this context of a potential “immunity gap,” our retrospective study analyzed hospitalized children with RSV or HRV. This study revealed that while HRV was the most frequently detected virus annually, RSV exhibited a sharp seasonal peak in winter, which was driven almost exclusively by the RSV-A subtype. In addition, RSV infection resulted in more severe acute illness (higher rates of fever, cough, and pneumonia and longer hospitalization), whereas HRV infection was characterized by a distinct eosinophil-predominant inflammatory profile. Finally, multivariable analysis identified both HRV infection and elevated eosinophil counts as independently associated with the subsequent development of asthma even after accounting for the strong influence of prior wheezing history.

The epidemiological patterns observed in our study reflect the impact of strict NPIs on viral ecology. Consistent with prior reports [19, 20], RSV accounted for a substantial proportion of hospitalized patients in our cohort (58.9%, 425/722). We observed that RSV activity was highly concentrated in a sharp seasonal peak during late 2022 (November–December), predominantly affecting infants (median age 0.77 years). This intense winter circulation, accounting for a significant portion of annual hospitalizations, likely resulted from rapid transmission through an immunologically naive population accumulated due to the “immunity gap” [21]. RSV has been classified into subgroups A and B on the basis of genetic and immunological analyses and they are reported to take dominance alternately, with a major prevalent pattern of “BBAA” most of the time in China [22, 23]. A previous Shanghai study confirmed the prevalence of RSV-B from October 2019 to October 2021, marking the “BB” phase [24]. Our finding of overwhelming RSV-A dominance in 2022 (96.8% of subtyped cases) suggests a marked subtype shift during the study period. However, because whole-genome sequencing was not performed, the mechanisms underlying the observed RSV-A predominance cannot be determined in the present study. Post-NPI population susceptibility may have influenced viral circulation, but possible explanations such as founder effects, differences in transmissibility, or circulation of specific lineages including ON1 remain speculative and require confirmation in future molecular epidemiological studies. In contrast to RSV's concentrated seasonality, HRV circulated year-round and exhibited a bimodal distribution. This pattern is consistent with previous reports and suggests that HRV, as a nonenveloped virus, possesses greater environmental resilience [25].

Our analysis revealed different clinical and laboratory profiles between RSV and HRV infections, which reflect distinct underlying pathophysiological processes. Patients in the RSV group had a greater incidence of fever, a longer duration of both fever and cough, and a significantly greater rate of progression to pneumonia than did those in the HRV group (*P* < .05). This aligns with the established pathology of RSV, which causes extensive damage to the small airway epithelium, leading to more pronounced lower respiratory tract signs [26, 27]. In contrast to some reports suggesting that RSV infection in young infants tends to be afebrile [28, 29], our data revealed fever in 61.9% of RSV cases, although the median duration was short. This may be influenced by specific virus strains, the age of the infected patient, and the immune status of the host.

Our findings indicated a clear divergence in the host response to HRV and RSV. While RSV infection was associated with greater acute morbidity, such as pneumonia, HRV infection triggered a distinct systemic inflammatory profile characterized by significant leukocytosis (*P* < .001). More importantly, HRV infection was associated with significantly higher eosinophil counts, which is consistent with the findings of a previous study [30, 31]. The median eosinophil count in HRV patients was more than four times greater than that in RSV patients. These findings are consistent with, but do not directly establish, a type 2-skewed inflammatory pattern during HRV infection. Because no cytokine profiling or airway inflammatory assessment was performed in this study, the underlying immunological mechanisms cannot be inferred directly from our data. Furthermore, because preinfection blood counts were not available, it is not possible to determine whether the observed eosinophilia was induced by HRV infection itself or reflected a preexisting eosinophilic predisposition in children who were subsequently hospitalized with HRV. Prior studies have linked HRV-associated eosinophilia to type 2 immune responses and altered antiviral pathways [32, 33], but these mechanisms remain interpretive in the context of the present study. Therefore, this apparent paradox, where the acutely more severe pathogen (RSV) differs from the one associated with a pro-asthmatic inflammatory signature (HRV), prompted us to investigate which of these initial influences truly predicts long-term asthma development.

To resolve the paradox of whether acute severity or a specific inflammatory signature predicts long-term morbidity, we analyzed the subsequent incidence of asthma. Our Kaplan–Meier analysis revealed a significantly greater rate of asthma development in the HRV group than in both the RSV and coinfection groups (log-rank *P* < .001). This finding aligns with landmark prospective cohorts such as the COAST study, which identified early life HRV infection as the most significant viral precursor to childhood asthma [34]. However, the link between early viral infections and subsequent asthma remains debated in some studies. Kusel et al reported that RSV and HRV infections in the first year after birth were associated with wheezing and asthma at the age of 5 years, with no difference between the 2 groups [35]. Uppala R. et al reported that specific pathogens (RSV/HRV) did not account for a statistically significant difference in subsequent wheezing or asthma development [36]. Our results support the hypothesis that HRV infection is more strongly associated with asthma inception than RSV infection in this specific post-NPI population. However, this finding should be interpreted with caution. Children in the HRV group were older and had higher prevalences of atopy, family history of atopy, and prior wheezing, which may indicate a greater baseline susceptibility to later asthma.

Our multivariable regression analysis further showed that, even after adjustment for prior wheezing history and other measured covariates, both HRV infection (aOR 1.85, 95% CI: 1.15–2.99) and the eosinophil count (aOR 2.43, 95% CI: 1.07–5.49) remained independently associated with subsequent asthma diagnosis. These findings indicate that the observed association with later asthma was related not only to baseline susceptibility but also to viral etiology and the inflammatory profile present during acute infection. However, because preinfection eosinophil counts were not available, we cannot determine whether the elevated eosinophil levels observed during HRV infection were a consequence of the infection or a preexisting host characteristic that predisposed children to both HRV-related hospitalization and subsequent asthma. This finding is consistent with established immunological findings where HRV, unlike RSV, is strongly associated with the production of IL-5, the key cytokine for eosinophil proliferation and survival [37]. Although both RSV and HRV can induce IL-8 and IFN-λ production, prior studies suggest that RSV infection tends to elicit a relatively greater IL-8 and IFN-λ response, which is more closely linked to acute neutrophilic inflammation and epithelial damage than to chronic allergic airway remodeling [38–40].

Notably, our analysis also revealed distinct risk profiles for asthma and recurrent wheezing. While HRV infection can predict both outcomes, specific host factors differ. An elevated eosinophil count and a prior history of wheezing were specific risk factors only for a formal asthma diagnosis, suggesting that progression to a stable asthmatic phenotype requires specific underlying eosinophilic inflammation or cumulative airway injury. In contrast, younger age emerged as a significant risk factor, specifically for recurrent wheezing (aOR 0.86, *P* = .045), possibly reflecting the anatomical vulnerability of the narrower airways in younger infants to transient wheezing during viral infections. Therefore, Therefore, while recurrent wheezing in the youngest children may be largely explained by airway immaturity, progression to a formal asthma diagnosis appears to be more closely linked to the combination of HRV infection and eosinophilic inflammation, whether this eosinophilia represents a preexisting host trait, a virus-induced response, or both.

This study has several limitations. First, baseline differences between the RSV and HRV groups, including age, prior wheezing history, and atopic or family history, may have introduced residual confounding despite multivariable adjustment. Second, reverse causation cannot be excluded, as children predisposed to asthma may have been more likely to be hospitalized with HRV infection. Thus, this retrospective observational study cannot determine whether HRV contributes directly to asthma pathogenesis or preferentially affects children already at higher risk. Third, the single center design may limit generalizability. Fourth, most asthma diagnoses occurred before 4 years of age and were based on physician diagnosis reported at follow-up, so some preschool wheezing disorders may have been classified as asthma [41]. Fifth, peripheral blood counts were only indirect markers of airway inflammation, and neither whole genome sequencing nor HRV subtyping was performed. Finally, although the routine multiplex assay covered a broader respiratory pathogen panel, the study database was restricted to children with RSV and or HRV positivity, and other pathogens were not analyzed at the hospital wide level. Despite these limitations, the large sample and consistent associations provide useful evidence on early respiratory risk.

## CONCLUSION

In 2022, during the final phase of strict NPIs in China, our study highlighted the distinct impacts of the RSV and HRV. Although RSV infection caused greater acute severity, HRV infection demonstrated a stronger independent association with subsequent childhood asthma. This association, together with the eosinophilic inflammatory profile observed during HRV infection, persisted after adjustment for the child's prior wheezing history. However, whether the eosinophilia observed at the time of HRV infection reflects a virus-induced response or a preexisting host predisposition cannot be determined from the current study design and warrants investigation in future prospective studies with preinfection baseline measurements. We also observed a shift in viral ecology, with a near-exclusive dominance of RSV-A during this period. Consequently, clinical risk stratification for childhood asthma should integrate viral etiology and inflammatory markers alongside patient history.

## Supplementary Material

ofag314_Supplementary_Data

## Supplementary Data

Supplementary materials are available at *Open Forum Infectious Diseases* online. Consisting of data provided by the authors to benefit the reader, the posted materials are not copyedited and are the sole responsibility of the authors, so questions or comments should be addressed to the corresponding author.
