# Supplementary material for: Acute Severity Versus Long-term Morbidity: Uncoupling the Roles of RSV and HRV in Childhood Respiratory Disease
Source: Open Forum Infect Dis. 2026 May 21;13(6):ofag314. doi: 10.1093/ofid/ofag314 (PMC13232741; doi:10.1093/ofid/ofag314)
Supplement: ofag314_Supplementary_Data [file ofag314_supplementary_data.zip › Supplement 2 Follow-up questionnaire.docx]

Supplement 2 Follow-up questionnaire for recurrent wheezing and asthma

ID. Gender Age Date of follow-up

1. Has your child experienced any episode of wheezing since discharge from the hospital?

A. Yes B. No

2.If yes, can you identify any trigger(s) for the wheezing episode(s)? (multiple selections allowed)

A. Respiratory infection   B. Allergen exposure   C. Exercise   D. Unknown E. Other: __________

3. How many wheezing episodes has your child experienced in the past 12 months?

A. < 3 episodes   B. ≥ 3 episodes

4. Has your child been diagnosed with asthma by a pediatrician during the follow-up period?

A. Yes B. No

5. If yes, at what age was the diagnosis made? __________

6. Has any other significant respiratory event (e.g., emergency department visit or hospitalization for wheezing) occurred since discharge?

A. Yes (please specify): __________   B. No
