# Supplementary material for: Acute Severity Versus Long-term Morbidity: Uncoupling the Roles of RSV and HRV in Childhood Respiratory Disease
Source: Open Forum Infect Dis. 2026 May 21;13(6):ofag314. doi: 10.1093/ofid/ofag314 (PMC13232741; doi:10.1093/ofid/ofag314)
Supplement: ofag314_Supplementary_Data [file ofag314_supplementary_data.zip › Supplement Table 1.docx]

Supplement Table 1. Comparison of available baseline characteristics between the source population and the final analytic cohort.

| Characteristics | Source population (N = 2,393) | Analytic cohort (N = 722) |
| --- | --- | --- |
| Viral group, n (%) |  |  |
| RSV only | 886 (37.0%) | 425 (58.9%) |
| HRV only | 1,479 (61.8%) | 275 (38.1%) |
| Co-infection | 28 (1.2%) | 22 (3.0%) |
| Sex, n (%) |  |  |
| Male | 1,440 (60.2%) | 444 (61.5%) |
| Female | 953 (39.8%) | 278 (38.5%) |
| Age |  |  |
| Age (years), median (IQR) | 2.08 (3.41) | 1.08 (2.80) |
| < 28 days, n (%) | 65 (2.7%) | 44 (6.1%) |
| 28 days to < 1 year, n (%) | 713 (29.8%) | 297 (41.1%) |
| 1 to < 6 years, n (%) | 1,303 (54.5%) | 372 (51.5%) |
| ≥ 6 years, n (%) | 312 (13.0%) | 9 (1.2%) |
| Seasonal distribution, n (%) |  |  |
| January to June | 784 (32.8%) | 151 (20.9%) |
| July to December | 1,609 (67.2%) | 571 (79.1%) |

Note: Data for the source population were extracted from the general laboratory surveillance database. Age text strings from the raw database were converted into decimal years for accurate calculation and grouping. Detailed clinical information, full laboratory parameters, atopic history, and follow up data were collected exclusively for the final analytic cohort.
